# The Nurse and the Nation

**Published:** 1920-03-06

**Authors:** 


### THE NURSE AND THE NATION.

PURSUING my remarks of last week concerning the College elections, I want to warn the reader in advance that I am afraid I am going to skate on very thin ice. And I want at the same time to ask those whose feelings I may shock to give some serious consideration to my suggestions, as they are the outcome of serious thought, not to say strong conviction.

What I have to say is directly counter to the shibboleth that nurses are perfectly well able to manage their own affairs. I know it is heresy, and that I am not only taking risks myself, but I am asking my Editor to share them. Shall I awake to find that THE HOSPITAL is ordered to be burnt by the common hangman?

I have no evidence that the nurse is able to manage her own affairs. I have advanced even further than this in the danger zone. I do not believe she wants to be troubled with managing them. When her day's toil is done she has not the time, the energy, or the inclination. The "affairs" to which I refer are many and complex, and their successful management demands qualities and talents which very few nurses possess. Englishmen flatter themselves that they manage their own affairs. Do they? "I hae ma doots." They have a Welshman managing a good many of them, and English women are now going to have a finger in the pie!

I hold the belief, and the conviction grows stronger every day, that the British nurse will not come into her heritage until the British public are aroused. I am a whole-hearted believer in British fair play, but I realise how hard it is to get the British ear. The receiver is usually off when you call, and there is no answer.

Do not be impatient. I am coming to the point. By way of illustration: I had occasion the other day to ring up an exalted person, and I might as well have been signalling Mars. And after weary hours of failure I chanced on a friend who listened to my tale of woe. "Ring up Club X," he said; "he is there now, for I have just left him." "No," I said; "you ring him up and give him my message. He knows me only by repute, if he knows me at all. You can dig him in the ribs."

The whole business was transacted in ten minutes. Cannot nurses learn from this how to set about catching the ear of the public? This is what they need to do, as I hope to demonstrate with force in the early future.

The ear of the British public has, so far, been reached in a casual way only. There has been a word dropped in by the way, and between times. If you desire to verify this, get a bundle of hospital reports, and search for appeals on behalf of the nurse. When you find them, cut out the paragraphs, paste them on a sheet of paper, and send them hither. I tried this experiment, and, lo! my sheet is blank.

The message of the nurse to the British public has not been delivered. The Postmaster-General of the nurse is her College, and it is of the highest importance that the nurse's call should be heard.

Hence I venture to express the hope that the College will see to it, when the elections come, amongst the nominees will be included at least one public woman of outstanding personality and force of character. I mean by this the type of woman who would be an asset of much national value if she were a Member of Parliament. It would be invidious and probably unseemly for me to mention names. In truth, I have no name in my mind. I am discussing a policy which appears to me to be worth general and deep consideration, and I put it to matron and nurse—Is it not eminently desirable that the College of Nursing should be represented in Parliament by a woman, or by more than one?

The nurse is not a private individual. She is a public servant of inestimable value. Her vocation appeals to the noblest men and women in the land, from the Royal Family downwards, and quite recently the Prince of Wales was appointed President of the oldest hospital in the Kingdom.

"Hitch your wagon to a star!" and do not be misled by suggestions which, although, no doubt, they are honestly conceived, lead downwards. If you want to dilute the Executive of matrons, do so by all means, but not by electing candidate matrons. What you want is to catch the ear of the nation. This can only be done by using the proper speaking-trumpet. We are on the eve of a great National Health campaign, and it is imperative that the nurse should be permitted to participate. She is immeasurably the best of all possible candidates, and, judging by the latest evidence before me, which I propose to discuss in the early future, the nurse runs imminent risk of being superseded.

Hence the urgent necessity of vigorous action. I appeal to the College Executive if this is good common-sense or otherwise.

IERNE.

